# The association between breastfeeding and childhood obesity: a meta-analysis

**DOI:** 10.1186/1471-2458-14-1267

**Published:** 2014-12-13

**Authors:** Jing Yan, Lin Liu, Yun Zhu, Guowei Huang, Peizhong Peter Wang

**Affiliations:** Department of Social Medicine and Health Administration, School of Public Health, Tianjin Medical University, Tianjin, China; Division of Community Health and Humanities, Faculty of Medicine, Memorial University of Newfoundland, St. John’s, Newfoundland and Labrador A1B 3 V6 Canada; Department of Nutrition and Food Science, School of Public Health, Tianjin Medical University, Tianjin, 300070 China; School of Public Health, Tianjin Medical University, Tianjin, China

**Keywords:** Breastfeeding, Children, Obesity, Meta-analysis

## Abstract

**Background:**

The increase in childhood obesity is a serious public health concern. Several studies have indicated that breastfed children have a lower risk of childhood obesity than those who were not breastfed, while other studies have provided conflicting evidence. The objective of this meta-analysis was to investigate the association between breastfeeding and the risk of childhood obesity.

**Methods:**

The PubMed, EMBASE and CINAHL Plus with Full Text databases were systematically searched from start date to 1^st^ August 2014. Based on the meta-analysis, pooled adjusted odds ratio (AOR) and 95% confidence interval (CI) were calculated. I^2^ statistic was used to evaluate the between-study heterogeneity. Funnel plots and Fail-safe N were used to assess publication bias and reliability of results, and results from both Egger test and Begg test were reported.

**Results:**

Twenty-five studies with a total of 226,508 participants were included in this meta-analysis. The studies’ publication dates ranged from 1997 to 2014, and they examined the population of 12 countries. Results showed that breastfeeding was associated with a significantly reduced risk of obesity in children (AOR = 0.78; 95% CI: 0.74, 0.81). Categorical analysis of 17 studies revealed a dose-response effect between breastfeeding duration and reduced risk of childhood obesity.

**Conclusion:**

Results of our meta-analysis suggest that breastfeeding is a significant protective factor against obesity in children.

## Background

Obesity has become a major public health crisis around the world. Since overweight and obesity are strongly correlated with diseases such as diabetes, hypertension, high cholesterol, cardiovascular diseases, stroke, arthritis, and certain types of cancers, the number of obesity-related death is estimated at 2.8 million per year [1, 2]. Childhood obesity has also dramatically worsened and is now considered a major threat to human health [3–5]. According to the International Obesity Taskforce (IOTF) [1] and the World Health Organization (WHO) [6, 7], there are more than 200 million overweight or obese children throughout the world and, in 2010, over 42 million children under age of 5 were classified as overweight. Unfortunately, childhood obesity is linked to several physical and mental health conditions, including orthopedic problems, menstruation problems, sleep trouble, depression, anxiety and diabetes in childhood [8, 9]. Moreover, overweight/obese children are more likely to be obese in adulthood [10].

Childhood obesity has multiple causes, including genetic factors, personal behaviors (e.g., exercise, sleep duration, and TV viewing), dietary habits, and their interactions [11–14], and many researchers have investigated various possible interventions to prevent childhood obesity. Among these, breastfeeding has been associated with a decreased risk of obesity, along with other health benefits for the child and mother. According to the WHO recommendations, infants should be exclusively breastfed for the first 6 months, and breastfeeding should be supplemented with additional foods for the first 2 years (or beyond) [15]. Breast milk is considered the ideal food for infants, as it provides adequate energy and nutrients to meet the infants’ needs. In addition, as breast milk is safe and contains antibodies, breastfeeding could reduce the risk of neonatal infection, gastrointestinal infection, and pneumonia during infancy [9, 10]. It has been indicated that approximately 45% of neonatal infectious deaths, 30% of diarrhoeal death and 18% of respiratory death among children <5 years old are associated with suboptimal breastfeeding [15]. Moreover, breastfeeding has long-term benefits throughout a child’s lifetime. Children and adults who were breastfed have lower rates of overweight/obese, type-2 diabetes, hypertension, and are known score higher on intelligence tests than persons who were formula-fed [12, 15]. Based on the WHO report, if every child in the world was exclusively breastfed for the first 6 months, followed by breastfeeding until 2 years, the lives of 800,000 children would be saved each year [16]. Additionally, breastfeeding protects mothers against breast cancer, ovarian cancer, and obesity. Exclusive breastfeeding also is an effective, natural method of birth control, providing 98% protection between birth and 6 months postpartum [15].

In 2012, approximately 38% of infants who were under 6 months old were exclusively breastfed worldwide [15], with additional feeding methods including partial breastfeeding and exclusively formula feeding [9, 12]. The major factors that affect prevalence and duration of breastfeeding include maternal race/ethnicity, education, breast diseases, inadequate breast milk production, employment, length of maternity leave, inadequate knowledge regarding breastfeeding, lack of familial and societal support, and lack of guidance and encouragement from health care professionals [9, 15]. To strengthen breastfeeding practices, families, employers, professional workers and society as a whole should fully support to breastfeeding mothers.

Over the previous decade, a growing body of research has indicated an association between breastfeeding and childhood obesity. Breastfeeding has been identified as a protective factor for childhood obesity in many studies [16–20], while other studies failed to establish an association between the breastfeeding and childhood obesity [21, 22]. Three previous reviews have addressed this issue, the latest one being published about 9 years ago in 2005 [23–25]. Arenz *et al.* [23] included 9 studies that focused on the relationship between breastfeeding and childhood obesity with adjusted odds ratio (AOR). Owen *et al.* [24] presented the pooled odds ratio (OR) for 28 studies reporting the effects of infant feeding on the risk of obesity, as well as the pooled AOR for 6 studies that adjusted for socioeconomic status, parental body mass index (BMI), and maternal smoking. Lastly, Harder *et al.* analyzed the relationship between the duration of the breastfeeding and overweight based on 17 studies [25].

Given the lack of recent reviews, we conducted a systematic review to assess the association between breastfeeding and childhood obesity, and to explore the nature of the association. Compared to the previous reviews [23–25], this analysis is different in both eligibility criteria and study methodologies. We reviewed all studies that published until 1^st^ August 2014 to generate an updated and extended data set and used AOR and 95% confidence interval (CI) to control for potential confounders. Moreover, the prevalence of breastfeeding is changing, and there are unique trends emerging in different countries. For example, the prevalence of breastfeeding is increasing in the UK [26], while the prevalence of exclusive breastfeeding is declining in China [27]. Thus, this review provides important updated data to reflect the changing of breastfeeding throughout the world.

## Methods

### Literature search

The search strategy was comprised of the following steps: 1) formulation of the main topic, and inclusion and exclusion criteria; and 2) literature search, including both electronic databases, hand-search of key journals (e.g., Obesity, International Journal of Obesity, Pediatric Obesity, International Journal of Pediatric Obesity) and the references from the retrieved papers. We systematically searched the following databases: PubMed, EMBASE and CINAHL Plus with Full Text databases. The following keywords were used: (breastfed*) AND (obesity OR overweight OR adiposity) AND (child* OR infant). The publication language was restricted to English and Chinese, and the publication date was up to 1^st^ August 2014 with no lower date limit. All retrieved articles were screened according to pre-defined inclusion and exclusion criteria (described below) by two authors. Any disagreement was resolved in discussion with the project lead.

### Study selection

Studies were included if they fulfilled the following eligibility criteria: evaluated the relationship between breastfeeding and obesity; provided AOR with 95% CI for the association of obesity with breastfeeding; reported potential confounding factors, defined obesity and/or overweight, and breastfeeding type; and included study subjects older than 1 year. Studies were excluded on the basis of the following criteria: focused on other disease; were duplicates; provided incomplete data (e.g., no AOR, 95% CI) or insufficient data for calculation of these estimates; did not provide data on BMI; were not published as full text; were not primary research (e.g., reviews, commentaries, consultants’ corners, letters, conference abstracts). If the same study sample was used in 2 or more studies, findings of the largest sample size or the longest follow-up periods were included. Two investigators independently completed the literature search and selection procedures. If a discrepancy occurred regarding article selection, the 2 authors would discuss or refer to other authors.

### Data extraction

A standardized form was used to extract data independently by 2 authors. The following information from each article was extracted: first author, publication year, study design, study population characteristics of country, ethnicity, age, feeding patterns (e.g., never-ever breastfeeding, breastfeeding duration), BMI, sample size, and data provided such as AOR or data used to calculate the AOR, corresponding 95% CI, and confounding factors (e.g., birth weight, gender, age, maternal overweight, maternal smoking, maternal education, socioeconomic status, dietary habits, exercise). Disagreements between the 2 authors were resolved by discussion or referred to other authors.

### Obesity and breastfeeding definition

We used BMI, which was calculated by dividing the individual’s weight (in kilograms) by the square of their height (in meters), as an index of obesity [28]. As there were no standard BMI cutoffs to label obesity or overweight in children, the definitions of obesity were not uniform across eligible studies. The IOTF defined overweight or obesity as BMI ≥25 kg/m^2^ or ≥30 kg/m^2^ [28–30]; several studies used a BMI percentiles as the cutoff values of obesity, including BMI ≥95^th^ or BMI ≥97^th^ percentile [29]; and other variables to define the cutoff, for example, BMI ≥90^th^, BMI ≥94^th^, BMI >2 standard deviations (SD), and Chinese children’s BMI cutoffs of obesity (Boys’ BMI ≥96.3^th^, Girls’ BMI ≥98^th^). The calculation of BMI for child and adolescent population was the same as that for adults; however, childhood obesity (age under 20 years) was defined by comparing with children of the same sex and age [28, 29]. Given these diverse criteria for obesity, we considered whether they might affect the accuracy of our estimate, and conducted sensitivity analysis to evaluate the influence.

Data were categorized according to various breastfeeding variables, and information regarding breastfeeding was typically obtained from the parents. Several studies grouped infants into “ever breastfed” versus “never breastfed”, or “exclusively breastfed” versus “mixed fed” versus “exclusively formula fed”. Ever breastfeeding was defined as any attempt at breastfeeding, even if only for a short time; never breastfeeding was defined as no breastfeeding; exclusively breastfeeding was defined as breastfeeding without supplementation (e.g., no solid food, tea, herbal preparation or liquids); mixed feeding was defined as a combination of breastfeeding and formula feeding; and exclusively formula feeding was defined as only formula feeding [15, 23]. Other studies classified children in terms of the duration of breastfeeding, which was measured in weeks or months. Therefore, we stratified the included articles for the subgroup analysis as never-ever breastfeeding (i.e., ever breastfed versus never breastfed, exclusively breastfed versus exclusively formula fed) and breastfeeding duration. Studies using exclusive breastfeeding versus mixed feeding versus exclusive formula feeding were grouped into the never-ever breastfeeding category.

### Statistical analysis

Based on the aforementioned eligibility criteria, data on AOR and 95% CI were extracted from each included study. We calculated the I^2^ statistics (0% ~ 100%) to explain the between-study heterogeneity, with I^2^ ≤ 25% suggesting more homogeneity, 25% < I^2^ ≤ 75% suggesting moderate heterogeneity, and I^2^ > 75% suggesting high heterogeneity [31, 32]. If the null hypothesis was rejected, a random effects model was used to calculate pooled effect estimates [33]. If the null hypothesis was not rejected, a fixed effects model was used to calculate pooled effect estimates [33]. Sensitivity analysis was performed to assess how results vary by study design, definitions of obesity and breastfeeding, type of breastfeeding, and adjustment for potential confounding factors. Publication bias was assessed first with the funnel plot [34, 35], followed by formal statistical tests. The funnel plot graphically checked the existence of publication bias in meta-analyses. Egger test and Begg test were reported with a *p* < 0.05 being considered statistically significant, in order to avoid limitations of the funnel plot [36–38]. Reliability of results were examined for each job satisfaction predictor though Fail-safe N. All analyses were performed using Comprehensive Meta Analysis Version 2.2.064, provided by Biostat.

## Results

### Study selection and characteristics

**Figure 1:**
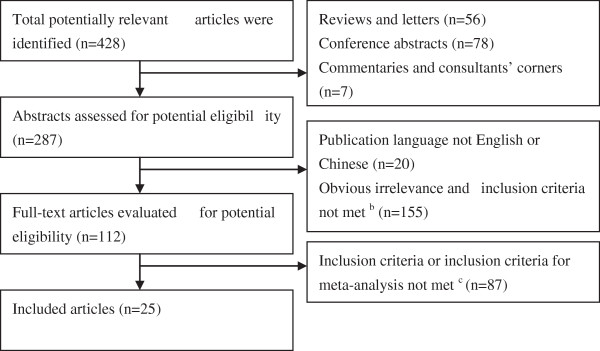
**Flowchart of literature search and selection process** ^**a**^ **.** ^a^Please contact the corresponding author for detailed search strategy. ^b^The study did not focus on childhood obesity, breastfeeding, or the relationship between breastfeeding and childhood obesity. The study provided incomplete data. ^c^The study provided incomplete data. The weight was not expressed as BMI.

The comprehensive literature search of electronic databases, key journals, and cross-references yielded 718 publications, which included 428 unduplicated articles, regarding the association between obesity and breastfeeding published before 1^st^ August 2014 as potentially relevant articles. A total of 25 studies with 226,508 subjects [39–63] were included in the present meta-analysis. The publication dates for these studies ranged from 1997 to 2014, and involved the population of 12 countries, including 5 German studies, 5 American studies, 3 British studies, 3 Australian studies, 2 Chinese studies, 1 Japanese study, 1 Irish study, 1 Greek study, 1 Brazilian study, 1 Dutch study, 1 Czech study, and 1 Canadian study. Of these, 24 included studies published in English and 1 study in Chinese. Ten studies were cross-sectional surveys and 15 were cohort studies, which included 10 prospective cohort studies and 5 indicate historical cohort studies. The selection process is detailed in Figure 1.

The description of the included studies is provided in Table 1. Six of the 25 studies defined overweight and obesity according to the IOTF recommendation [45, 47, 49, 52, 57, 58], 10 studies defined overweight as BMI ≥85^th^ percentile and obesity as BMI ≥95^th^ percentile according to the criteria developed by the Centers of Disease Control and Prevention (CDC) [39, 42–44, 46, 50, 53, 54, 60, 61], 5 studies defined overweight and obesity according to the German reference as BMI ≥90^th^ percentile and ≥97^th^ percentile [40, 41, 48, 59, 62], and 4 studies defined obesity alternatively as BMI ≥90^th^ percentile, BMI ≥94^th^ percentile, BMI >2 SD, and the Chinese children’s BMI cut-off for obesity [51, 55, 56, 63].

**Table 1:** **Characteristics of included studies in the meta-analysis**

Reference	Origin	Study design	Age	Sample size ^a^	Definition of feeding patterns	Definition of obesity	Confounders
Armstrong 2002 [39]	Great Britain	IHC	39-42 months	32,200	Formula-fed, BF	BMI ≥ 95^th^	Gender, birth weight, SES
Bergmann 2003 [40]	Germany	PC	6 years	480	Bottle-fed, BF	BMI ≥ 97^th^	Overweight mother, SES^c^
Beyerlein 2008 [41]	Germany	CS	54-88 months	9,368	Never & ever BF	BMI ≥ 97^th^	Weight gain from birth to years of life, maternal BMI, parents’ graduate^bc^
Burke 2005 [42]	Australia	PC	1-8 years	1,672	Never, BF ≤ 4 mo, BF ≤ 8 mo, BF ≤ 12 mo, BF > 12 mo	BMI ≥ 95^th^	Birth weight, gestational age, ethnicity, sex, maternal BMI, first child, and maternal education^c^
Gillman 2001 [43]	United States	CS	9-14 years	14,377	Mostly or only fed formula, mostly or only BF	BMI ≥ 95^th^	Age, sex, birth weight, birth order, Tanner stage, exercises, daily energy intake, mother’s BMI, and household income, dietary restraint, weight cycling, and weight concerns^bc^
Grummer-Strawn 2004 [44]	United States	IHC	4 years	12,587	Never, BF < 1 mo, BF ≤ 2.9 mo, BF ≤ 5.9 mo, BF ≤ 11.9 mo, BF ≥ 12 mo	BMI ≥ 95^th^	Gender, race/ethnicity, birth weight, and mother’s age, education, prepregnancy BMI, and weight gain during pregnancy ^c^
Hawkins 2009 [45]	Great Britain	PC	3 years	13,172	Never, BF < 4 mo, BF ≥ 4 mo	IOTF	Birth weight, gender, ethnicity, introduction of solid foods, maternal SES, education, lone motherhood status, age at first live birth, number of children, household income, parental overweight, country, et al.^bc^
Hediger 2001 [46]	United States	CS	3-5 years	2,656	Never & ever EBF	BMI ≥ 95^th^	Birth weight, race/ethnicity, gender, age group, mother’s BMI, timing of introduction to solid foods
Jwa 2014 [47]	Japan	PC	1.5-8 years	41,572	Formula feeding, mixed feeding, EBF	IOTF	Birth weight, having elder sibling(s), maternal educational level, age and total house-hold income at 0.5 years old ^c^
Koletzko 2009 [48]	Germany	CS	5-6 years	9,357	Never & ever BF	BMI ≥ 97^th^	Parental education, low birth weight, own bedroom, frequent consumption of butter^c^
Kwok 2010 [49]	Hong Kong	PC	7 years	7,026	Never, partially or EBF <3 mo, EBF ≥ 3 mo	IOTF	Sex, birth weight, gestational age, birth order, mother’s age at birth, birthplace, number of hospital admissions at 0–3 months, parental education, occupation, income^c^
Li 2003 [50]	Great Britain	CS	4-18 years	2,631	Never, BF ≤ 1 mo, BF ≤ 3 mo, BF ≤ 6 mo, BF ≤ 9 mo, BF > 9 mo	BMI ≥ 95^th^	Sex, parent’s BMI, birth weight, and social class^c^
Liese 2001 [51]	Germany	CS	9-10 years	2,108	Formula-fed, BF	BMI > 90^th^	Age, gender, city, nationality, SES, number of siblings^c^
McCrory 2012 [52]	Republic of Ireland	IHC	9 years	7,798	Never, BF ≤ 4 wk, BF ≤ 8 wk, BF ≤ 12 wk, BF ≤ 25 wk, BF ≥ 26 wk	IOTF	Dietary quality, exercise, maternal education, gestational age, nationality, weight status, household income^bc^
Moschonis 2008 [53]	Greek	IHC	1-5 years	2,374	Exclusive formula-fed, mixed, EBF	BMI ≥ 95^th^	Parental education, weight status, maternal gestational diabetes mellitus, alcohol consumption patterns during pregnancy, age at birth, birth rank^c^
Moss 2014 [54]	United States	PC	2-4 years	14,150	Never & ever BF	BMI ≥ 95^th^	Maternal education, maternal age, maternal race, family poverty, children’s birth weight and gender
Novaes 2011 [55]	Brazilian	CS	6-10 years	764	Never & ever BF	BMI > 2SD	Gender, physical education classes, siblings, maternal nutritional state during pregnancy^bc^
O’Challaghan 1997 [56]	Australia	PC	5 years	2,034	Formula fed, BF ≥ 6 mo, exclusiveness unclear	BMI ≥ 94^th^	Birth weight, gander, small for gestational age, feeding problems, sleeplessness, parental BMI, maternal education, household income
Scholtens 2008 [57]	Dutch	PC	8 years	2,043	Never, BF ≤ 16 wk, BF > 16 wk	IOTF	Maternal education, overweight, diet at 7 years of age^bc^
Scott 2012 [58]	Australia	IHC	9-16 years	2,066	Never, BF < 2 mo, BF < 4 mo, BF < 6 mo, BF ≥ 6 mo	IOTF	Age, gender, energy intake, physical activity, sleep duration, maternal age, education, ethnicity^b^
Toschke 2002 [59]	Czech Republic	CS	6-14 years	33,768	Never & ever BF	BMI ≥ 97^th^	Parental education and obesity, birth weight, sports outside school, number of siblings^bc^
Twells 2010 [60]	Canada	CS	4 years	1,026	formula feeding ≥ 3 mo, mixed feeding ≥3 mo, EBF ≥ 3 mo	BMI ≥ 95^th^	Birth weight, gender, age, maternal education, whether the baby was preterm or full term^c^
Van Rossem 2010 [61]	United States	PC	3 years	884	Never, any BF <6 mo, partial BF ≥ 6 mo, EBF ≥ 6 mo	BMI ≥ 95^th^	Age, sex, maternal educational, race, BMI, pregnancy weight gain, birth weight, gestational age^c^
Von Kries 1999 [62]	Germany	CS	5-6 years	9,206	Never, EBF ≤ 2 mo, EBF ≤ 5 mo, EBF ≤ 12 mo, BF > 12 mo	BMI ≥ 97^th^	Parental education, low birth weight, own bedroom, consumption of butter^c^
Zhao 2013 [63]	China	PC	2-8.4 years	1,189	Formula feeding, mixed feeding, EBF	Boys’ BMI ≥96.3^th^ Girls’ BMI ≥98^th^	Maternal weight and BMI before pregnancy, maternal weight increase and the level of blood glucose during pregnancy, gender, birth weight, father weight

*Abbreviation*: *IHC* indicates historical cohort, *PC* Prospective cohort, *CS* Cross-sectional, *BMI* body mass index (calculated as weight in kilograms divided by height in meters squared), *BF* breastfeeding, *EBF* Exclusive breastfeeding, *IOTF* the International Obesity Task Force, *SES* socio-economic status.

^a^The number of participants included in the analysis with different feeding patterns at last follow-up.

^b^Adjustment for amount of watching TV for children.

^c^Adjustment for maternal smoking in pregnancy.

### Main meta-analysis results

**Figure 2:**
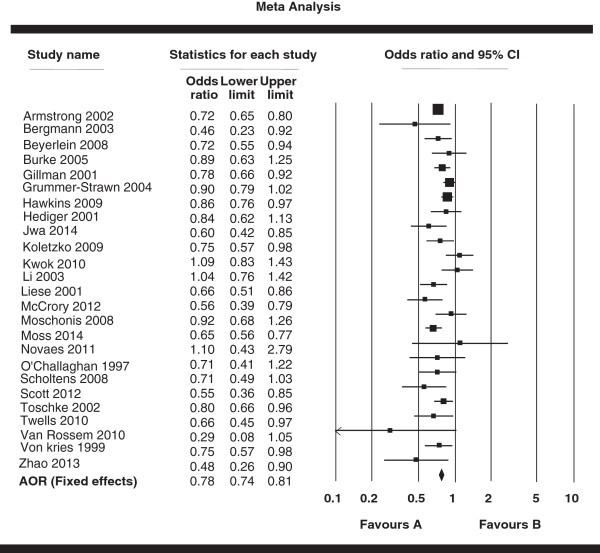
**Forest plot of the associations between breastfeeding (never & ever) and obesity in all 25 studies.** Adjusted odds ratios and 95% confidence intervals.

**Figure 3:**
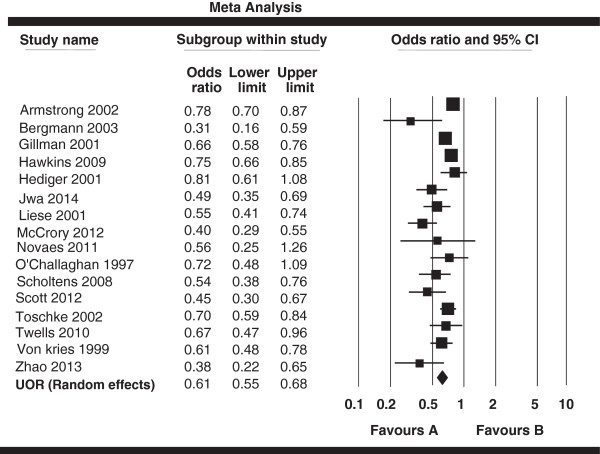
**Forest plot of the associations between breastfeeding (never & ever) and obesity in 16 studies.** Unadjusted odds ratios (UOR) and 95% confidence intervals (CI).

The pooled AOR derived from all 25 studies was 0.78 (95% CI: 0.74, 0.81) and the details are presented in a Forest plot (Figure 2). There was no significant heterogeneity across studies (*I*^*2*^ = 45.28%, *P* < 0.01), and the studies met fixed effects model. Additionally, we analyzed unadjusted data of results. Sixteen studies [39, 40, 43, 45–47, 51, 52, 55–60, 62, 63] presented information on the crude ORs, and the pooled crude OR and the corresponding 95% CIs are shown in Figure 3 with a forest plot. The pooled OR was 0.61 (95% CI: 0.55, 0.68). The homogeneity hypothesis was rejected (*I*^*2*^ = 65.40%, *P* < 0.01), and hence the studies met random effected meta-analysis criteria.

### Sensitivity analysis

Sensitivity analysis was carried out to evaluate differences in study design, definitions of obesity and breastfeeding, type of breastfeeding, and adjustment for potential confounding factors (Table 2). Findings suggested that studies that classified children using the breastfeeding duration showed a more protective effect of breastfeeding against childhood obesity than other studies. The 6 studies that applied the IOTF definition of obesity, showed weaker associations between breastfeeding and childhood obesity, than studies that defined childhood obesity as BMI ≥95^th^, BMI ≥97^th^ or other standards. Moreover, there was no difference between the pooled estimates across cohort studies and cross-sectional studies. Additionally, our results showed slightly effects of breastfeeding type, and whether the data were adjusted for the confounders, amount of watching TV and exercise in children. In addition, we performed a sensitivity analysis by removing 1 study, and found that the pooled AOR remained largely unchanged (AOR = 0.78, 95% CI: 0.74, 0.81).

**Table 2:** **Findings for subgroup analyses of breastfeeding and childhood obesity**

Component	No. of studies (sample size ^a^)	AOR (95% CI) fixed-effected
Study design		
Cohort	15 (141,247)	0.78 (0.73,0.82)
Cross-sectional	10 (85,261)	0.78 (0.72,0.84)
Whether consider amount of TV viewing and exercise of children as adjusted confounders		
Yes	8 (83,356)	0.79 (0.73,0.85)
No	17 (143,152)	0.77 (0.73,0.82)
Obesity definition		
IOTF	6 (73,677)	0.81 (0.73, 0.89)
BMI ≥ 95^th^	10 (84,557)	0.78 (0.74, 0.83)
BMI ≥ 97^th^	5 (62,179)	0.75 (0.67, 0.85)
Others	4 (6,095)	0.66 (0.53, 0.82)
Breastfeeding definition		
Never-ever	11 (126,512)	0.80 (0.76, 0.85)
Exposure: breastfeeding duration	14 (99,996)	0.73 (0.67, 0.79)
Breastfeeding type		
Exclusive breastfeeding	8 (65,933)	0.80 (0.71, 0.90)
Others	17 (160,575)	0.77 (0.74, 0.81)

*Abbreviation*: *CI* confidence interval, *AOR* adjusted odds ratio.

^a^The total number of subjects for included studies in the analysis at last follow-up.

### Publication bias

**Figure 4:**
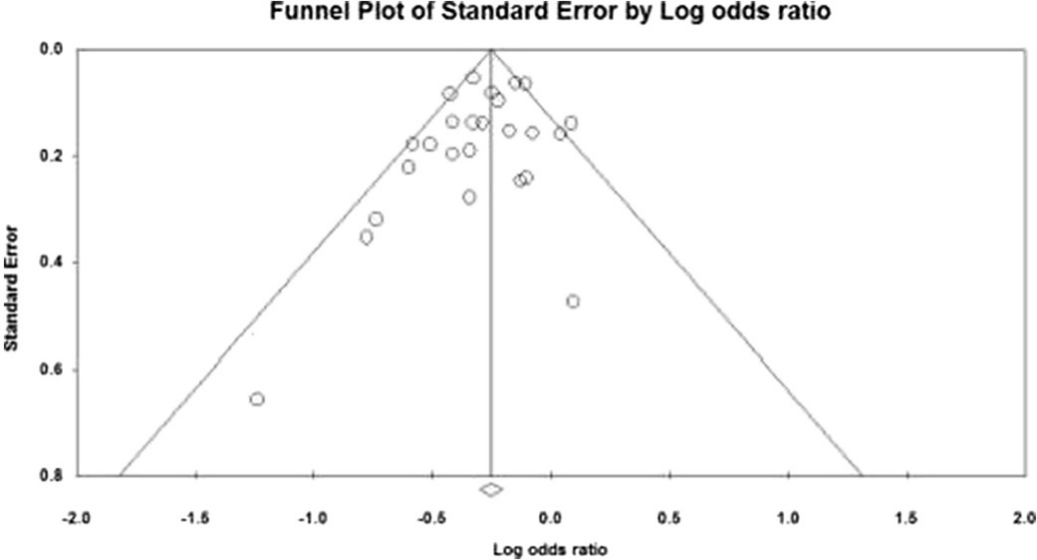
**Funnel plot of standard error by log odds ratio for meta-analysis of studies.**

The funnel plot presented an asymmetric pattern (Figure 4), and the results of Egger test (p = 0.16) and Begg test (p = 0.25) were not statistical significant. The result of Fail-safe N (N_fs0.05_ = 939.78) indicated high reliability of results. Therefore, we cannot completely exclude potential publication bias.

### Breastfeeding duration

Seventeen studies analyzed the association between breastfeeding duration and obesity, and 9 of these studies presented 2 or more categories for breastfeeding durations. A total of 51 estimates included in the meta-analysis were categorized as <3 months, 3–5 months, 5–7 months and ≥7 months (Table 3). The length of breastfeeding period was associated with a decreased risk of childhood obesity. Children breastfed for ≥7 months were significantly less likely to be obese (AOR = 0.79, 95% CI: 0.70, 0.88), while those breastfed for <3 months showed about 10% decrease in the risk of childhood obesity. In addition, we observed a stepwise gradient of decreasing risk of obesity with increasing duration of breastfeeding, indicating a change to dose-response effect.

**Table 3:** **Associations between breastfeeding duration and childhood obesity**

Breastfeeding duration	No. of studies	AOR (95% CI) fixed-effected
<3 months	16	0.90 (0.84, 0.95)
3-4.9 months	8	0.88 (0.79, 0.97)
5-6.9 months	9	0.83 (0.76, 0.90)
≥7 months	18	0.79 (0.70, 0.88)

*Abbreviation*: *CI* confidence interval, *AOR* adjusted odds ratio.

## Discussion

The association between breastfeeding and childhood obesity has long been under debate; however, no agreement has been achieved with respect to this issue. This meta-analysis suggests that breastfeeding protects against obesity in childhood. The risk of childhood obesity was lower in breastfed children by 22% compared with those who were never breastfed. Meanwhile, a stronger link was found between breastfeeding and obesity based on the crude OR. Two previous meta-analyses have demonstrated that breastfeeding is a protective factor for obesity, but reported inconsistent pooled ORs [23, 24]. A change to dose-response effect between breastfeeding duration and childhood obesity was confirmed in our study, in agreement with Harder *et al.* [25]. In particular, breastfeeding for more than 7 months significantly decreased the risk of obesity [64].

This meta-analysis shows that breastfeeding is a protective factor of adiposity, in contrast to results from several studies, which have shown no link between breastfeeding and obesity [21, 22, 49]. There are several possible explanations for this finding. First, breast milk provides a moderate amount of calories and nutrients for infant, such as sugar, water, protein and fat [9]. Moreover, the composition of breast milk changes with time and the mother’s diet [65]. In contrast, formula feeding provides higher levels of fat and protein than the baby’s needs [66]. Higher protein and fat intake in early childhood have been associated with adiposity [67]. Moreover, breast milk rather than other milk contains bioactive substances such as leptin and ghrelin, which can influence the proliferation and differentiation of the infant’s adipocytes [52, 67]. Thus, breast milk is rich in effective ingredient with higher nutritional value.

Interestingly, many investigations have indicated that breast milk can be influenced by environmental and behavioral factors [66, 67]. However, several researchers have suggested that unadjusted results should not be ignored either irrespective of the adjusted results [68]. In the present analysis, we compared the pooled ORs before and after combined adjustment for confounders, and observed a decreasing trend after the adjustment for potential confounders. These results support the hypothesis that maternal and individual factors, including genetic, environmental, and behavioral factors, can influence the body shape of children [45, 69]. Nevertheless, the interaction among multiple factors is still unclear. Factors such as physical activity and screen time (e.g., computer use or TV viewing) are known to significantly affect on the risk of obesity among children [52, 55]. The subgroup analyses of our study show that breastfeeding was associated with a lower risk of obesity, and the association was independent of TV viewing and exercise level [24]. Therefore, the mechanism(s) by which breastfeeding influences the later life influences the risk of obesity appear to be complex [70].

In addition, stratified analyses revealed differences in obesity definition and breastfeeding definition in the studies we examined. However, it is important to note the smaller sample size in several categories, which may have reduced the statistical power [23, 24]. The subgroup analyses showed that the association between breastfeeding and childhood obesity was particularly strong in 4 studies that applied other definitions of obesity. The pooled estimates from cross-sectional studies, cohort studies, and studies defining obesity cutoff ≥ 95^th^ were similar to the overall pooled AOR; the pooled estimates from studies using other categories for subgroup analyses were also close to the overall pooled AOR. Moreover, the link between breastfeeding and childhood obesity was slightly different in studies that used different breastfeeding types. This may be because the original data from several studies did not distinguish between exclusive and partial breastfeeding, thereby reducing the precision of our analysis [57, 58]. Therefore, future studies should compare the results for exclusive breastfeeding, mixed feeding, and exclusive formula feeding.

A change to dose-response relationship between breastfeeding duration and childhood obesity was confirmed in this study [25]. Breastfeeding for <3 months provided a minor protective effect for childhood obesity, while breastfeeding for ≥7 months showed a significantly high protection. These results are inconsistent with the findings of several studies. For example, Toschke *et al.* reported no association between breastfeeding for <6 months and obesity [71]. Similarly, another study found children who were exclusively breastfed for <4 months did not have a reduced the risk of obesity [72]. In contrast, McCrory *et al.* performed a change to dose-response relationship in children breastfed for ≥4 weeks and lower risk of obesity [52], and another study showed breastfeeding for 1–16 weeks was associated with a significantly reduced risk of obesity [57]. These discrepancies in the findings may be related to the diverse populations (with unique genetic and environmental backgrounds) and different sample sizes.

This meta-analysis has several strengths. First, it was based on adjusted ORs and 95% CIs from each study, which largely ruled out residual confounding by other factors and improved the accuracy of the effect estimate. In addition, we analyzed the most recently data published (before 1^st^ August 2014) and historical data, with no lower date limit. Compared to previous reviews [23, 24], which reported the pooled AOR, this meta-analysis included a larger sample size, which improved the statistical power. Also, several limitations of this study should be considered. An important limitation is the funnel plot presented an asymmetry; thus, the publication bias cannot be fully ruled out. There are 2 possible explanations for this result. First, among the 25 included studies, 24 were published in English and 1 in Chinese, while other language were not included in this study. It is well accepted that studies showing significant results are more likely to be published, and more likely to be published in English, which is a common cause of publication bias. Although we searched several electronic databases of Chinese to increase the number of studies reported in Chinese in this analysis, none could be added due to the poor quality of reporting. Only 2 of the 25 included papers investigated the Chinese population, and the results from 1 study showed no association between breastfeeding and childhood obesity [49]. Therefore, confirmation of these findings is needed in further research conducted in the Chinese population. Second, we did not consider unpublished investigations that may lead to publication bias. Another limitation of the present analysis is the AORs of each study that were adjusted for different confounders. As well, a weaker association between breastfeeding and obesity was observed after the adjustment of known confounders. Therefore, if we adjust for the same and more relevant confounders, the protective effect of early breastfeeding might reduce largely. However, breastfeeding has been suggested to be a protective factor to childhood obesity in numerous studies [64, 73]. Moreover, because several studies did not report the breastfeeding type, we could not distinguish all the collected data between exclusive and partial breastfeeding, and hence, the precision of effect estimates may be influenced. However, the pooled estimate for exclusive breastfeeding was calculated, based on data from 8 studies [46, 47, 49, 53, 60–63]. Finally, the different BMI cut-offs using for defining obesity in different studies may also have had an impact on the overall estimate; therefore, we performed a subgroup analysis by obesity definition to minimize this limitation.

## Conclusion

In summary, the aim of this systematic review was to investigate the association between breastfeeding and childhood obesity. The results indicate a protective effect of breastfeeding for childhood obesity, and prolonged breastfeeding is directly related to a decreasing risk of obesity. In particularly, children being breastfed for ≥7 are significantly less likely to be obese in later childhood. Future research should distinguish the data between exclusive breastfeeding, mixed feeding, and exclusive formula feeding, and use uniform potential confounders.

## Authors’ information

PPW is a professor of epidemiology in the Faculty of Medicine of Memorial University of Newfoundland (MUN). GWH is a professor of dietetics/nutrition, and the Dean of the School of Public Health in Tianjin Medical University (TMU). LL and ZC are current Master’s students at MUN, while JY is a lecturer at TMU.

## Abbreviations

IOTF: International Obesity Task Force
WHO: World Health Organization
AOR: Adjusted odds ratio
OR: Odds ratio
BMI: Body mass index
CI: Confidence interval
SD: Standard deviations
CDC: Centers of disease control and prevention
IHC: Indicates historical cohort
PC: Prospective cohort
CS: Cross-sectional
BF: Breastfeeding
EBF: Exclusive breastfeeding
SES: Socio-economic status
UOR: Unadjusted odds ratios.
